# Co-occurrence of Primary Headache and Tremor in a Young Woman: A Case Report

**DOI:** 10.7759/cureus.93509

**Published:** 2025-09-29

**Authors:** Koji Hayashi, Yuka Nakaya, Mamiko Sato, Toyoaki Miura, Yasutaka Kobayashi

**Affiliations:** 1 Department of Rehabilitation Medicine, Fukui General Hospital, Fukui, JPN; 2 Graduate School of Health Science, Fukui Health Science University, Fukui, JPN

**Keywords:** hand tremor, headache, involuntary limb movement, positional tremor, primary headache disorder, rest tremor

## Abstract

This case report describes a 28-year-old woman presenting with right temporal headache and tremor of the right hand. She had a history of menstrual-related, nonthrobbing headaches but no prior tremors or visual symptoms. Neurological examination revealed a 5-Hz resting and postural tremor in the right upper limb, hyperreflexia, and normal cognitive function, with unremarkable magnetic resonance imaging findings. Blood tests showed euthyroidism and elevated inflammatory and lipid markers. Surface electromyography confirmed the tremor. The patient was diagnosed with tremor associated with primary headache and responded rapidly to clonazepam, with symptom resolution the next day. This case underscores the diagnostic difficulties presented by the atypical co-occurrence of primary headache and tremor. It highlights the importance of considering functional disorders in the differential diagnosis when encountering such presentations, alongside a thorough exclusion of organic causes.

## Introduction

Primary headaches are idiopathic pain conditions that represent one of the most common disorders of the nervous system and are significant contributors to disability [1]. Unlike secondary headaches, which arise from serious underlying pathologies, primary headaches are not caused by another medical condition [1]. Most headaches seen in emergency departments are primary in nature; however, it is crucial initially to screen for "red flags" that might indicate life-threatening secondary etiologies [1]. The main types of primary headaches include tension-type headaches (TTHs), migraines, cluster headaches, and exertional headaches [1,2].

Tremor is a common movement disorder characterized by involuntary, rhythmic oscillations around a joint, significantly impairing daily activities [3-5]. It is classified based on activation condition, anatomical distribution, and frequency [3,4]. Rest tremor occurs when the affected limb is relaxed and unsupported, as in Parkinson's disease, and tends to diminish with voluntary movement [3,5]. Action tremors, which occur during voluntary activity, include postural, kinetic, intention, task-specific, and isometric subtypes [3,5]. Anatomically, tremors most frequently affect the hands and arms, although distributions vary depending on the disorder [3]. Frequencies range from very low (<4 Hz) to very high (>12 Hz), further aiding in tremor classification [3,4].

Headache and tremor are both common neurological symptoms, but they are typically regarded as distinct clinical disorders. Their co-occurrence, however, is recognized in certain contexts, although the nature of the association often remains a subject of debate. For instance, a potential link between migraine and essential tremor (ET) has been investigated, with studies yielding conflicting results [6-11]. The simultaneous presentation of these symptoms can also be attributed to other known entities, such as head tremor associated with cervical dystonia, side effects of medications like tacrolimus or antidepressants, or as a manifestation of substance use and various underlying systemic or intracranial pathologies [8-10,12,13].

When the co-occurrence of primary headache and tremor does not fit into these established categories, it presents a significant diagnostic challenge for clinicians. The differential diagnosis in such cases is broad and requires careful exclusion of a wide range of etiologies, including functional neurological disorders. Furthermore, the pathophysiological mechanisms underlying these atypical presentations remain poorly understood.

Here, we present a rare case of a young woman who developed a primary headache accompanied by bilateral tremors of about 5 Hz. The tremors occurred both at rest and during sustained postures and voluntary movement, highlighting an unusual clinical overlap that does not align with previously reported etiologies. This case underscores the diagnostic challenges posed by such atypical presentations and aims to deepen our understanding of the potential neurophysiological interactions between pain and movement disorders.

## Case presentation

A 28-year-old woman presented to our department with a new-onset right-sided temporal headache and a concurrent tremor of her right hand. Both symptoms developed upon waking and lasted for several hours. Her headache was described as nonthrobbing and sharp, with a severity of 6 out of 10 on the Visual Analog Scale. They were not associated with nausea, vomiting, photophobia, or phonophobia. Although she had a history of menstrual-related, nonthrobbing headaches occurring approximately once a month since adolescence, typically milder and effectively managed with over-the-counter (OTC) analgesics, she described this headache as being different in character from her previous episodes. Her last menstrual period started 10 days prior and ended six days before presentation. She denied any history of scintillating scotomas (photopsia). Her mother also had a history of headaches responsive to OTC medication. The patient was not taking any regular medications known to cause tremors, apart from occasional herbal medicine (Kamishoyosan) for dysmenorrhea, and she denied use of caffeine, alcohol, or illicit substances.

On examination, she was alert with intact cognitive function. Neurological assessment revealed no cranial nerve abnormalities, limb weakness, or sensory disturbances. A 5-Hz tremor was observed in her right upper limbs, predominantly in the right forearm. The tremor was present at rest as well as during sustained postures and voluntary movement (Video 1). While observing the tremor, she noticed that a tremor which was not initially present had also appeared (but mild) in her left forearm. She had difficulty voluntarily suppressing the tremor, but it subsided somewhat when she diverted her attention; conversely, the tremor amplitude increased when she was the focus of others' attention. Furthermore, no subtle dystonic postures were observed in the hand or neck. Deep tendon reflexes were brisk bilaterally, but no pathological reflexes were noted. A detailed family history revealed no relatives with tremor, making ET less likely. Routine blood tests were significant only for a slightly elevated platelet count and total protein, with a normal thyroid-stimulating hormone level, confirming euthyroid status (Table 1).

**Video 1:** The patient's tremor on admission

**Table 1:** The blood test results on admission

Inspection items	Result	Reference range
Red blood cell	460 × 10⁴/μL	386-492 × 10⁴/μL
White blood cell	7,800/μL	3,300-8,600/μL
Hemoglobin	13.4 g/dL	11.6-14.8 g/dL
Platelet	41.9 × 10⁴/μL	15.8-34.8 × 10⁴/μL
Blood glucose	87 mg/dL	73-109 mg/dL
Glycated hemoglobin (HbA1c)	6.7%	4.9-6.0%
Total protein	8.2 g/dL	6.6-8.1 g/dL
Uric acid	4.5 mg/dL	2.6-7.0 mg/dL
Total bilirubin	0.3 mg/dL	0.4-1.2 mg/dL
Aspartate aminotransferase	18 U/L	13-30 U/L
Alanine aminotransferase	16 U/L	7-30 U/L
Alkaline phosphatase	78 U/L	38-113 U/L
Lactate dehydrogenase	200 U/L	124-222 U/L
γ-Glutamyltransferase	17 U/L	9-32 U/L
Creatine phosphokinase	84 U/L	41-153 U/L
Cholinesterase	400 U/L	201-421 U/L
Amylase	107 U/L	44-132 U/L
Blood urea nitrogen	8.8 mg/dL	8.0-20.0 mg/dL
Creatinine	0.57 mg/dL	0.65-1.07 mg/dL
Sodium	140 mmol/L	138-145 mmol/L
Potassium	4.1 mmol/L	3.6-4.8 mmol/L
Chloride	101 mmol/L	101.0-108.0 mmol/L
C-reactive protein	0.12 mg/dL	0.0-0.14 mg/dL
Thyroid-stimulating hormone	2.300 μIU/mL	0.500-5.000 μIU/mL
Free T3	2.90 pg/mL	2.30-4.00 pg/mL
Free T4	1.21 ng/dL	0.90-1.70 ng/dL

**Figure 1:**
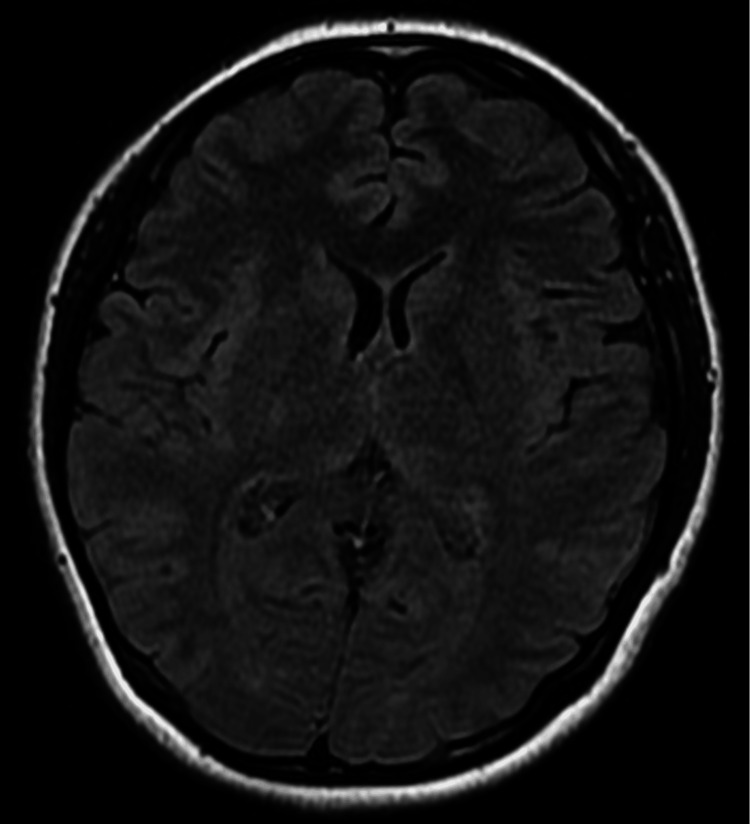
Brain MRI findings Brain MRI on T2-weighted FLAIR sequences showing no abnormalities, including in the basal ganglia MRI: magnetic resonance imaging; FLAIR: fluid-attenuated inversion recovery

**Figure 2:**
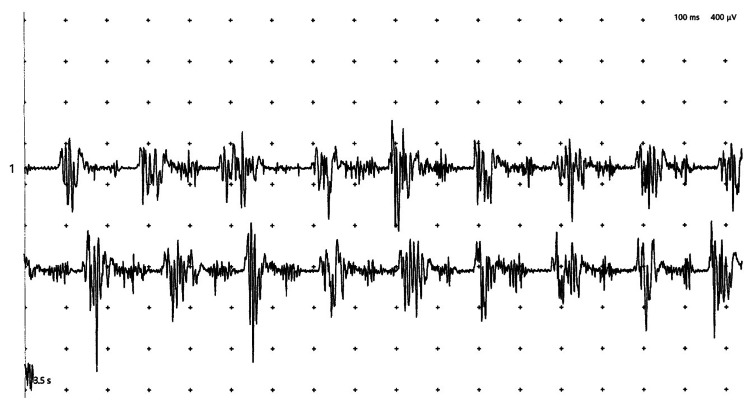
Surface electromyography results Surface electromyography was performed by placing electrodes on the flexor and extensor muscle groups of the elbow joint, which revealed a tremor with a frequency of approximately 5 Hz. The calibration for the recording is 100 ms per horizontal division and 400 µV per vertical division

Brain magnetic resonance imaging showed no abnormalities (Figure 1). Surface electromyography confirmed a regular 5-Hz tremor in the right forearm flexors and extensors (Figure 2). We suspected functional tremors, but given its regular rhythm and pattern and limited distractibility, the tremor's specific type remained undetermined. Additionally, we suspected TTH, but its specific type also could not be definitively determined. She was prescribed 0.5 mg of oral clonazepam, which resulted in complete resolution of both the tremor and headache by the following day. She had no further outpatient visits.

## Discussion

We present a rare case of a young woman experiencing a new-onset primary headache co-occurring with tremor. Her headache was unilateral, nonpulsatile, and without aura, and did not impair daily activities, so it did not meet criteria for migraine without aura [14]. It was bilateral, nonthrobbing, pressing, and lacked nausea, photophobia, or phonophobia, suggestive of probable TTH [14]. However, the diagnosis of definite TTH requires at least 10 episodes fulfilling specific criteria, including headache duration between 30 minutes and seven days, pressing quality, mild to moderate intensity, and absence of aggravation by routine physical activity, along with no more than one of photophobia or phonophobia [14]. Our patient's headache represented a new-onset single episode without sufficient frequency or duration to meet full diagnostic criteria. Therefore, while the clinical presentation aligns partly with probable TTH, it does not fulfill all required components for a definitive diagnosis per the International Classification of Headache Disorders, 3rd edition classification.

Regarding the tremors, we systematically considered several key possibilities. ET was initially considered due to the presence of a postural tremor component; however, the acute onset and the absence of a family history rendered this diagnosis less likely. Dystonic tremor was ruled out, given the lack of any subtle dystonic postures in the hand or neck during neurological examination. Functional tremor was a crucial consideration, particularly given the atypical presentation. Her tremor partially subsided with distraction and was exacerbated by observer attention, which might imply a variable tremor pattern and further support a functional etiology [15]. However, no fluctuations in tremor frequency were evident on surface electromyography. Furthermore, secondary causes for the tremor were diligently excluded: drug-induced tremor was ruled out based on her medication history, and normal thyroid function tests, along with a noncontributory brain magnetic resonance imaging (MRI), effectively eliminated major systemic or structural pathologies. Wilson's disease was considered highly unlikely given the patient's age, the absence of other neurological or hepatic signs, and a normal brain MRI; thus, specific laboratory testing was deemed unnecessary. Based on her clinical features and these considerations, we suspect both functional tremor and TTH, although a definitive diagnosis was not established.

The co-occurrence of headaches, especially migraine, and tremors has been observed clinically, but studies show conflicting results. Early research by Biary et al. reported a significant association with higher rates of migraine in ET patients and vice versa [6], whereas Barbanti et al. found no significant link [7]. Hu et al. later observed a higher prevalence of migraine in ET, suggesting population differences influence findings [8]. Kuiper et al. summarized that evidence remains inconsistent, partly due to age-related prevalence changes [9]. Recently, Fu et al. identified shared genetic mutation sites and found a correlation between migraine and tremor, with 23.96% of migraine patients exhibiting tremors [10].

Potential mechanisms for their coexistence include common responsiveness to beta-blockers like propranolol [6,9], neuroanatomical overlap, such as bilateral red nucleus activation on MRI [9], and abnormalities in the olivocerebellar circuit, which is implicated in ET and affected by cerebellar lesions in migraine [8,11]. Genetic factors, including the dopamine D3 receptor gene, expressed in key brain regions, may also contribute, although studies show mixed results regarding its role [8]. Additionally, shared genetic loci between migraine and dystonia may affect neurotransmitter release, with gamma-aminobutyric acid alterations implicated in both tremor and migraine [8,10].

Beyond the debated association between ET and migraine, several other conditions involve the co-occurrence of headache and tremor, each with distinct mechanisms. Head tremors from cervical dystonia and associated headaches have been described [16]. These headaches occur in the neck or back of the head, linked to dystonic muscles and upper cervical spine issues affecting the C1-C3 nerves [16]. This may contribute to migraine and cervicogenic headaches, as these nerves also innervate the dura and suboccipital muscles [16]. Dystonia can be subtle and often mistaken for ET. Nearly 37% of patients experience cervicogenic headache [16]. Additionally, drug-induced headache and tremor have been reported. Drug-induced headaches and tremors can be caused by medications like tacrolimus, which can cause neurotoxicity and tremor, especially at high blood levels [12]. Switching to extended-release formulas may reduce tremors [12]. Other drugs, such as antidepressants, mood stabilizers (like lithium), antiepileptics (like valproate and lamotrigine), and antiemetics (like metoclopramide), can also cause headaches and tremors as side effects [9]. Other etiologies for the co-occurrence of headache and tremor include substance abuse and withdrawal (such as alcohol and caffeine), drug-induced refractory headache, hyperthyroidism, hydrocephalus, space-occupying lesions (cysts and tumors), and intracranial infections (including West Nile virus, Listeria, and HIV) [9,13]. Additionally, several reports describe the co-occurrence of migraine with myoclonus or epilepsy [17,18], suggesting a nontrivial association between primary headaches and involuntary movements such as tremor.

In our case, the combination of headache and tremors, which were clinically diagnosed with TTH and functional tremor, differs from any of the above reports. She was treated empirically with clonazepam. The rapid and complete response to a single 0.5 mg dose of clonazepam is a noteworthy but diagnostically complex finding. Such a dramatic effect does not definitively distinguish between the differential diagnoses considered. While clonazepam can be effective for ET variants or some dystonic tremors through its GABAergic action, its potent anxiolytic properties can also produce a powerful therapeutic or placebo response in patients with functional tremor [15,19]. Therefore, while the treatment was successful, its diagnostic utility in this case is limited. The swiftness of the response could even be interpreted as lending further, albeit indirect, support to a functional component of the tremor, rather than confirming an organic etiology. Further studies are needed to elucidate the mechanism underlying this unique association.

A notable limitation is the absence of supportive clinical signs for functional tremors, such as entrainment and Hoover's sign [15], as well as neuropsychological testing. Had these indicators been present, they would have provided stronger evidence, enabling a more definitive and accurate diagnosis. Additionally, the patient did not return to our hospital, so follow-up data were not available. Had such data been available, the presentation would have been more informative.

## Conclusions

This report describes the case of a young woman with a co-occurrence of primary headache and tremor, where definitive etiologies like ET or migraine were not established. The clinical presentation highlighted significant diagnostic challenges, with features suggestive of both probable TTH and a functional tremor, though a definitive diagnosis remained elusive due to conflicting signs and limited follow-up data. This case illustrates the importance of a systematic approach to differential diagnosis in patients with atypical combinations of common neurological symptoms. Further reporting of similar cases is necessary to determine whether this co-occurrence represents a consistent clinical pattern or a coincidental finding.
